# Supplementary material for: Abnormal vasculature reduces overlap between drugs and oxygen in a tumour computational model: Implications for therapeutic efficacy
Source: PLoS Comput Biol. 2025 Dec 22;21(12):e1013801. doi: 10.1371/journal.pcbi.1013801 (PMC12758810; doi:10.1371/journal.pcbi.1013801)
Supplement: S1 Text — (PDF) [file pcbi.1013801.s001.pdf]

Suplementary information: Abnormal vasculature reduces overlap between drugs and oxygen in a tumour computational model: implications for therapeutic efficacy

Romain Enjalbert[1], Jakub Köry[2, †], Timm Krüger[3, a, *], Miguel O. Bernabeu[1, a, *]

1 Centre for Medical Informatics, Usher Institute, The University of Edinburgh, United Kingdom
2 School of Mathematics and Statistics, University of Glasgow, United Kingdom
3 School of Engineering, Institute for Multiscale Thermofluidics, The University of Edinburgh, United Kingdom
† Deceased May 2024
a Equally contributing senior authors
*** timm.krueger@ed.ac.uk, miguel.bernabeu@ed.ac.uk**

Blood flow model

Physical model

Blood is treated at each vessel segment as a one-dimensional continuous and incompressible fluid with an apparent viscosity, this is done through the use of Poiseuille's law [1]:

$$Q = \frac{\pi d_l^4 \Delta p}{128 L \mu_{\text{app}}}, \tag{1}$$

where Q is the flowrate of blood at each vessel segment, d_l is the vessel diameter of the vessel segment, Δp is the pressure drop along the vessel segment, L is the length of the vessel segment, and μ_{app} is the apparent viscosity of blood in the vessel segment. Poiseuille's law can also be expressed for an elliptical cross-section when vessels are compressed [2]:

$$Q = \frac{\pi a^3 b^3 \Delta p}{4 L \mu_{\text{app}} (a^2 + b^2)} \tag{2}$$

where a and b are the major and minor radius of the ellipse, respectively [2]. If $a = b$, the elliptical formulation of Poiseuille's law, Supplementary Eq. 2 is the same as the circular one, Supplementary Eq. 1.

The apparent viscosity of blood in each vessel segment is calculated using an empirically derived relationship where the apparent viscosity is dependent on vessel diameter and discharge haematocrit [3, 4]:

$$\mu_{\text{app}} = \mu_{\text{rel}} \mu_{\text{pl}} \tag{3}$$

where μ_{pl} is the apparent viscosity of pure plasma and μ_{rel} is the relative apparent viscosity, defined through

$$\mu_{rel} = 1 + (\mu_{45} - 1) \frac{(1 - H_D)^C - 1}{(1 - 0.45)^C - 1}, \tag{4}$$

where μ_{45} is the apparent viscosity of blood at a discharge haematocrit, H_D, of 45%, itself defined through the following relation

$$\mu_{45} = 220 e^{-1.3 d_l} + 3.2 - 2.44 e^{-0.06 d_l^{0.645}}, \tag{5}$$

where d_l is the diameter of the vessel segment in microns. C is defined through

$$C = (0.8 + e^{-0.075 d_l}) \Big(-1 + \frac{1}{1 + 10^{-11} d_l^{12}} \Big) + \frac{1}{1 + 10^{-11} d_l^{12}}. \tag{6}$$

This system of equations can be solved with known boundary conditions (either pressure or velocity boundary conditions) and if the discharge haematocrit is known at every vessel segment [5]. However, the discharge haematocrit is not known a priori, as red blood cells are distributed in networks disproportionately to blood flow [1]. The following relation allows one to predict the partitioning of red blood cells at each diverging microvascular bifurcation once the flowrates in the network are known

$$FQ_E = 0 \qquad \text{if } FQ_B \le X_0, \tag{7}$$

$$\text{logit}(FQ_E) = A + B\,\text{logit}\Big(\frac{FQ_B - X_0}{1 - 2X_0} \Big) \qquad \text{if } X_0 \le FQ_B \le 1 - X_0, \tag{8}$$

$$FQ_E = 1 \qquad \text{if } 1 - X_0 \le FQ_B, \tag{9}$$

$$\text{logit}(x) = \ln\Big(\frac{x}{1 - x} \Big), \tag{10}$$

where FQ_E is the flowrate fraction of red blood cells from the parent branch that flows to the daughter branch, and FQ_B is the fractional flowrate of blood from the parent branch that flows to the daughter branch. A, B, and X_0 are further defined by the following relationships

$$A = -13.29 \frac{\frac{D_\alpha^2}{D_\beta^2} - 1}{\frac{D_\alpha^2}{D_\beta^2} + 1} \frac{1 - H_D}{D_P}, \tag{11}$$

$$B = 1 + 6.98 \frac{1 - H_D}{D_P}, \tag{12}$$

$$X_0 = C \frac{1 - H_D}{D_P}, \tag{13}$$

where D_α is the diameter of the child branch for which FQ_E and FQ_B are calculated, D_β is the diameter of the other child branch, D_P is the parent branch diameter, and H_D is the parent branch discharge haematocrit. The value of C depends on whether the parent branch is compressed and $C = 0.96$ for circular (non-compressed) vessels [4], while $C = 4.16$ for compressed vessels with an aspect ratio of 4.26 [2], as assumed in this work. This leaves only the discharge haematocrit of inlets to be defined as a final boundary condition to solve the system of equations.

Numerical model

The physical model described above needs to be numerically solved. The solution is obtained through a previously described iterative scheme [5, 6], necessary due to the flow dependent disproportional partitioning of red blood cells in Supplementary Eqs. 7–9. At each iteration of the solver, the system of Poiseuille equations, Supplementary Eqs. 1–2, is solved with the known boundary conditions. With the known flowrate in each vessel, the discharge haematocrit is calculated in each vessel, Supplementary Eqs. 7–9, where the discharge haematocrit in the inlet vessels is also a fixed boundary condition. The solution for the flowrate of both blood and red blood cell flow at each iteration is compared to the solution at the previous iteration is then compared. The solver is considered to have converged when

$$\left| \frac{Q_{\mathrm{curr}} - Q_{\mathrm{prev}}}{Q_{\mathrm{curr}}} \right| \leq 0.00001 \tag{14}$$

where Q_{curr} and Q_{prev} are the values in the current and previous iteration of a given flowrate (blood or red blood cell) in a vessel. The iterative process continues until all vessels have converged.

The solver is implemented in a custom python2.7 code.

Network pruning

Fig Aa illustrates the original network that is generated. The Poiseuille solver that we use would have no flow in the dead-end vessels in the networks, so we recursively prune the dead-end vessels until there are none left, as illustrated in Fig Ab. This leads to avascular areas in the network, as shown by the black asterisk in Fig Ab, which are the result of the non-space filling of the vessels in two-dimensions. In addition, the blue asterisk in Fig Ab shows an example of an area where boundary effects would become important for the reaction-diffusion equations in the periphery region of the network.

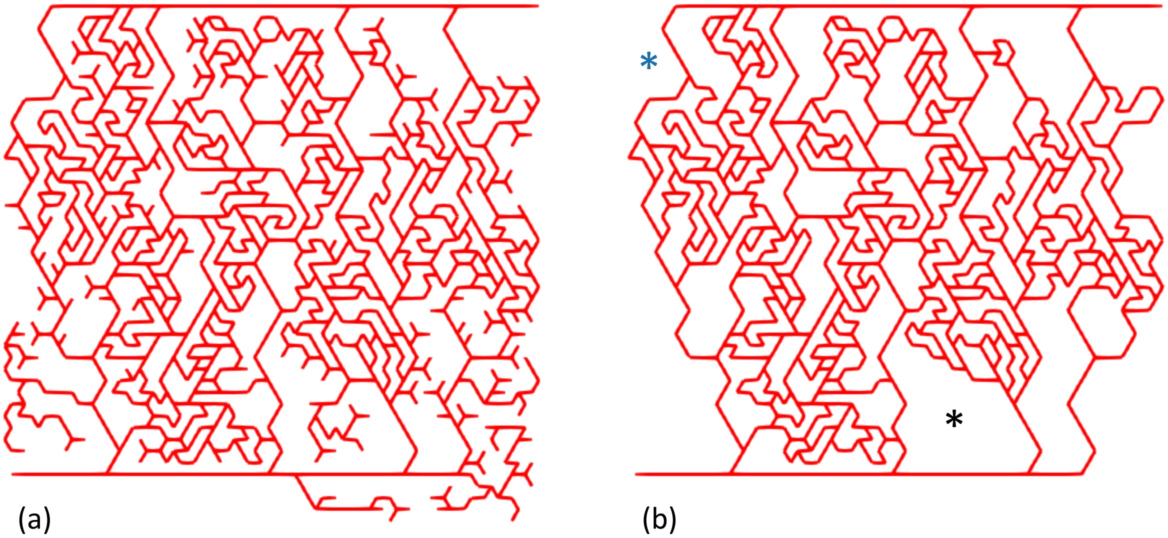

Fig A. (a) Shows the network generated by tumorcode [7]. (b) Shows the network with dead-end vessels pruned. Blue asterisk shows example of avascular tissue area due to boundary effect. Black asterisk shows example of avascular tissue area due to lack of connectivity in two-dimensions.

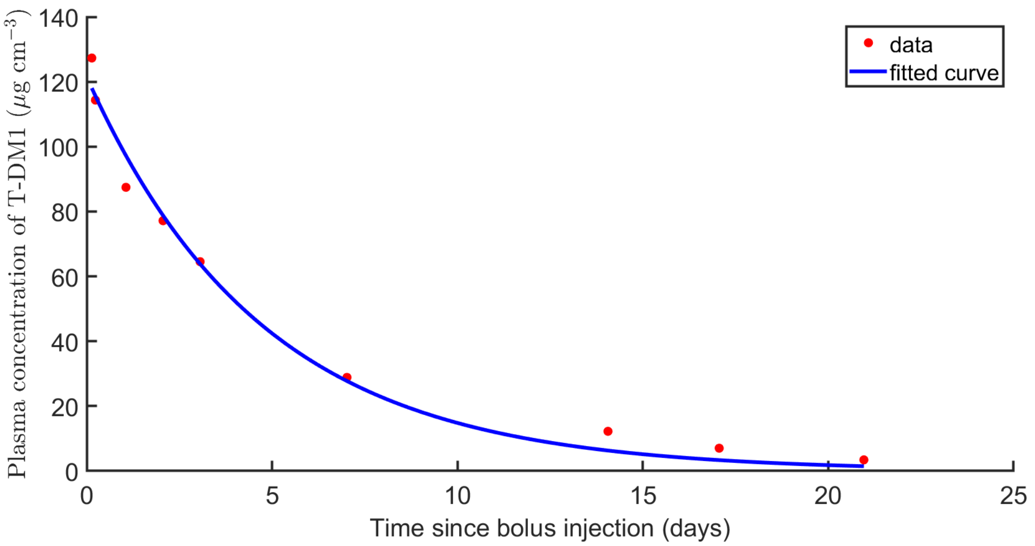

Fig B. Time evolution of plasma concentration using the highest-dose from [11] and the corresponding single-term exponential fit.

Table A. Parameters for T-DM1 simulations.

Parameter	Meaning	Value	Units	Source
k_{on}	Binding constant	9.49×10^2	$\text{M}^{-1}\ \text{s}^{-1}$	[8]
k_{off}	Unbinding rate	1.48×10^{-1}	s^{-1}	[8]
k_{int}	Internalization rate	8.80×10^{-4}	s^{-1}	[8]
D	Diffusivity in tissue	4.90×10^{-2}	$\mu\text{m}^2\ \text{s}^{-1}$	[8]
C_r	Receptor concentration	10^4	nM	[8]
ε	Void fraction	0.63	–	[8]
P	Vascular permeability	2.78×10^{-7}	cm s^{-1}	[10]
$C_{\text{plasma}}^{\text{initial}}$	Initial plasma concentration	810	nM	Estimated from [11]
b	Plasma depletion rate	0.2105	day^{-1}	Estimated from [11]

Reduced-order model for drug perfusion

Estimation of dimensional parameters

The majority of parameters governing pharmacokinetics of T-DM1 are readily available from [8]: see Table 1 (non-FUS fitted parameters) for the void fraction (referred to as "interstitium effective porosity") and Table 2 (non-FUS fitted parameters) for the other parameters. As a trastuzumab molecule is much larger than its emtansine counterpart (as measured by the respective molecular weights: 738 Da for DM1 and 145,167 Da for trastuzumab [9]), we estimate the vessel permeability for T-DM1 with the value for trastuzumab, i.e. $P = 10^{-3}$ cm h^{-1} $= 2.78 \times 10^{-7}$ cm s^{-1} [10]. To fully specify the model, we need to provide a relationship describing the depletion of T-DM1 from the blood stream, i.e. $C_{\text{plasma}}(t)$. To this end, we reproduce the highest-dose (4.8 mg/kg) data from Figure 1a in [11], using linear scaling for the Y axis and find the best single-term exponential fit to the data using MATLAB's fit() functionality. The best fit is shown in Fig B. The resulting single-term exponential fit is summarized in Eq. (3). Note that we used the molecular weight of T-DM1 from [9] ($\approx 1.5 \times 10^5$ Da, as discussed above) to convert the units (μg to mol); this way the fitted initial (for instance) plasma concentration of 121.5 μg cm^{-3} is converted to 8.1×10^{-10}mol cm^{-3} $= 810$ nM. All dimensional models parameters are summarized in Table A.

Nondimensionalization

We nondimensionalize concentrations with respect to the (constant) receptor concentration C_r, lengths with respect to a typical, for a randomly selected location in the tissue domain, distance to the nearest vessel $s_{\min}$ (here estimated as 50 μm) and time with respect to the plasma depletion timescale (the only slow timescale independent of locally-varying quantities – vessel diameter and inter-vessel distance), i.e.

$$C = C_r\tilde{C} \qquad B = C_r\tilde{B} \qquad I = C_r\tilde{I} \qquad C_{\text{plasma}} = C_r\tilde{C}_{\text{plasma}} \qquad (x,y) = s_{\min}(\tilde{x},\tilde{y}) \qquad t = \frac{1}{b}\tilde{t},$$

where the dimensionless quantities are denoted with tildes. Recalling that the units of Dirac delta function equals one over the units of its argument, we get from Eqs. (2) after algebraic manipulation the following dimensionless model

$$\begin{aligned}
\tilde{\alpha}_{\text{pd}}\frac{\partial\tilde{C}}{\partial\tilde{t}} &= \tilde{\alpha}_{\text{diff}}\tilde{\nabla}^2\tilde{C} + \tilde{\alpha}_{\text{ev}}\left(\varepsilon\tilde{C}_{\text{plasma}} - \tilde{C}\right)\tilde{\delta}_{\text{network}} - \tilde{C} + \tilde{\alpha}_{\text{off}}\tilde{B} \\
\tilde{\alpha}_{\text{pd}}\frac{\partial\tilde{B}}{\partial\tilde{t}} &= \tilde{C} - \tilde{\alpha}_{\text{off}}\tilde{B} - \tilde{\alpha}_{\text{int}}\tilde{B} \\
\tilde{\alpha}_{\text{pd}}\frac{\partial\tilde{I}}{\partial\tilde{t}} &= \tilde{\alpha}_{\text{int}}\tilde{B},
\end{aligned} \tag{15}$$

where

$$\begin{aligned}
&\tilde{\alpha}_{\text{pd}} = \frac{b\varepsilon}{k_{\text{on}}C_r} \approx 1.62\times10^{-4} \qquad \tilde{\alpha}_{\text{diff}} = \frac{D\varepsilon}{s_{\min}^2 k_{\text{on}}C_r} \approx 1.3\times10^{-3} \\
&5.56\times10^{-3} < \tilde{\alpha}_{\text{ev}}(d_l) = \frac{\pi d_l P}{s_{\min}^2 k_{\text{on}}C_r} < 4.08\times10^{-2} \qquad \tilde{\alpha}_{\text{off}} = \frac{k_{\text{off}}\varepsilon}{k_{\text{on}}C_r} \approx 9.83 \qquad \tilde{\alpha}_{\text{int}} = \frac{k_{\text{int}}\varepsilon}{k_{\text{on}}C_r} \approx 5.84\times10^{-2}
\end{aligned} \tag{16}$$

denote dimensionless parameter groupings indicating plasma-depletion, diffusion, extravasation, unbinding and internalization rates relative to the binding rate $k_{\text{on}}C_r/\varepsilon$. Note that these dimensionless groupings can alternatively be expressed in terms of standard dimensionless numbers governing pharmacokinetics, such as Biot and Damkohler numbers [12]. Finally, the dimensionless counterpart of the plasma depletion Eq. (3) reads

$$\tilde{C}_{\text{plasma}}(\tilde{t}) = \tilde{C}_{\text{plasma}}^{\text{initial}}e^{-\tilde{t}}, \qquad \text{where} \quad \tilde{C}_{\text{plasma}}^{\text{initial}} = \frac{C_{\text{plasma}}^{\text{initial}}}{C_r} \approx 8.1\times10^{-2}. \tag{17}$$

Model reduction

By considering the estimates in Supplementary Eq. (16), the time derivative in the second equation in Supplementary Eq. (15) can be neglected, yielding the first quasi-steady approximation

$$\tilde{B} = \frac{\tilde{C}}{\tilde{\alpha}_{\text{off}} + \tilde{\alpha}_{\text{int}}}, \tag{18}$$

which upon redimensionalizing gives Eq. (4). Substituting Supplementary Eq. (18) back into the first equation in Supplementary Eq. (15) gives

$$\tilde{\alpha}_{\text{pd}}\frac{\partial\tilde{C}}{\partial\tilde{t}} = \tilde{\alpha}_{\text{diff}}\tilde{\nabla}^2\tilde{C} + \tilde{\alpha}_{\text{ev}}\left(\varepsilon\tilde{C}_{\text{plasma}} - \tilde{C}\right)\tilde{\delta}_{\text{network}} - \frac{\tilde{\alpha}_{\text{int}}}{\tilde{\alpha}_{\text{int}} + \tilde{\alpha}_{\text{off}}}\tilde{C}.$$

From the estimated values in Supplementary Eq. (16), we get $\tilde{\alpha}_{\text{int}}/(\tilde{\alpha}_{\text{int}} + \tilde{\alpha}_{\text{off}}) \approx 5.91\times10^{-3}$ and we conclude that the time-derivative term can be neglected yielding the final reduced-order model

$$0 = \tilde{\alpha}_{\text{diff}}\tilde{\nabla}^2\tilde{C} + \tilde{\alpha}_{\text{ev}}\left(\varepsilon\tilde{C}_{\text{plasma}} - \tilde{C}\right)\tilde{\delta}_{\text{network}} - \frac{\tilde{\alpha}_{\text{int}}}{\tilde{\alpha}_{\text{int}} + \tilde{\alpha}_{\text{off}}}\tilde{C}.$$

Upon redimensionalizing, we arrive at Eq. (5) which is solved in Microvessel Chaste [13].

Simulations on simple geometry confirm the validity of the reduced-order model

Simple geometry: model and boundary conditions

To confirm the validity of above-derived approximation, we propose the same problem on a simple geometry containing only a single, infinitely-long, cylindrical blood vessel of diameter d_l with the axis oriented in the Z direction. This vessel supplies the drug to a surrounding tissue domain in the form of a cylindrical annulus of an inner diameter d_l and an outer diameter equal to an inter-vessel distance h_l, where both d_l and h_l cover ranges found in our networks. Provided no variation in drug concentration in the Z direction (i.e. along the vessel) is introduced via initial or boundary conditions, we can assume that the resulting drug profile remains axisymmetric, i.e. $C(t,x,y,z) = C(t,r)$, where $r = \sqrt{x^2+y^2}$ denotes the polar radius. Importantly, the Dirac delta source term in Eq. (2) is here replaced by the boundary condition

$$D\frac{\partial C}{\partial r} = -P\left(C_{\text{plasma}} - \frac{C}{\varepsilon}\right) \tag{19}$$

imposed at $r = d_l/2$ and we also impose zero-flux boundary condition

$$\frac{\partial C}{\partial r} = 0 \tag{20}$$

at $r = h_l/2$. The full model thus reads

$$\begin{aligned}
\frac{\partial C}{\partial t} &= \frac{D}{r}\frac{\partial}{\partial r}\left(r\frac{\partial C}{\partial r}\right) - k_{\text{on}}\frac{CC_r}{\varepsilon} + k_{\text{off}}B \\
\frac{\partial B}{\partial t} &= k_{\text{on}}\frac{CC_r}{\varepsilon} - k_{\text{off}}B - k_{\text{int}}B \\
\frac{\partial I}{\partial t} &= k_{\text{int}}B,
\end{aligned} \tag{21}$$

whereas the reduced-order model reads

$$0 = \frac{D}{r}\frac{\partial}{\partial r}\left(r\frac{\partial C}{\partial r}\right) - k_{\text{eff}}\,C, \tag{22}$$

where k_{eff} is defined in Eq. (5), supplemented by Eq. (4) and the third equation in Eq. (2) governing B and I, respectively. Both problems are to be solved on a spatial domain $d_l/2 \le r \le h_l/2$ subject to boundary conditions, Supplementary Eq. (19) and Supplementary Eq. (20), and the initial conditions

$$C(t=0,r) = 0 \qquad B(t=0,r) = 0 \qquad I(t=0,r) = 0. \tag{23}$$

Analytic solution of the reduced-order problem

Due to its linearity, the reduced-order problem can (when posed on the simple geometry) be solved directly. Multiplying Supplementary Eq. (22) by r^2 and dividing by D we get

$$r^2\frac{\partial^2 C}{\partial r^2} + r\frac{\partial C}{\partial r} - \frac{k_{\text{eff}}}{D}r^2 C = 0,$$

Denoting $s = \sqrt{k_{\text{eff}}/D}\; r$ and $F(t,s) = C(t,r)$, this problem transforms to

$$s^2\frac{\partial^2 F}{\partial s^2} + s\frac{\partial F}{\partial s} - s^2 F = 0 \tag{24}$$

which is to be solved in the domain $\sqrt{k_{\text{eff}}/D}\; d_l/2 \le s \le \sqrt{k_{\text{eff}}/D}\; h_l/2$, subject to boundary conditions

$$\frac{\partial F}{\partial s}\left(t, s=\sqrt{\frac{k_{\text{eff}}}{D}}\frac{d_l}{2}\right) = -\frac{P}{\sqrt{Dk_{\text{eff}}}}\left(C_{\text{plasma}}(t) - F\left(t, s=\sqrt{\frac{k_{\text{eff}}}{D}}\frac{d_l}{2}\right)/\varepsilon\right) \quad \text{and} \quad \frac{\partial F}{\partial s}\left(t, s=\sqrt{\frac{k_{\text{eff}}}{D}}\frac{h_l}{2}\right) = 0.$$

For any fixed time t, Supplementary Eq. (24) is a modified Bessel equation of order 0 and the solution is therefore a linear combination of its two linearly independent solutions $I_0(s)$ and $K_0(s)$ (modified Bessel functions of the first and second kind), i.e.

$$F(t,s) = F_1(t)I_0(s) + F_2(t)K_0(s). \tag{25}$$

Letting ' denote the derivative with respect to s, the two boundary conditions then give (for arbitrary fixed time t) a system of two linear equations for the unknown $F_1(t)$ and $F_2(t)$ of the form

$$I_0'\left(s = \sqrt{\frac{k_{\text{eff}}}{D}}\frac{h_l}{2}\right)F_1(t) + K_0'\left(s = \sqrt{\frac{k_{\text{eff}}}{D}}\frac{h_l}{2}\right)F_2(t) = 0$$

$$\left(I_0'\left(s = \sqrt{\frac{k_{\text{eff}}}{D}}\frac{d_l}{2}\right) - \frac{P}{\varepsilon\sqrt{Dk_{\text{eff}}}}I_0\left(s = \sqrt{\frac{k_{\text{eff}}}{D}}\frac{d_l}{2}\right)\right)F_1(t) +$$
$$\left(K_0'\left(s = \sqrt{\frac{k_{\text{eff}}}{D}}\frac{d_l}{2}\right) - \frac{P}{\varepsilon\sqrt{Dk_{\text{eff}}}}K_0\left(s = \sqrt{\frac{k_{\text{eff}}}{D}}\frac{d_l}{2}\right)\right)F_2(t) = -\frac{PC_{\text{plasma}}(t)}{\sqrt{Dk_{\text{eff}}}}$$

Finally, using known properties of modified Bessel functions, we have

$$I_0'(r) = I_1(r) \quad \text{and} \quad K_0'(r) = -K_1(r)$$

for any r. This system can easily be solved and $F_1(t)$ and $F_2(t)$ obtained for arbitrary $t > 0$.

Numerical method for the solution of the full model

Recall the full dimensional problem Supplementary Eq. (21) which is to be solved on a one-dimensional spatial domain $d_l/2 \le r \le h_l/2$ over time interval $0 \le t \le T$ (where $T = 21$ days), subject to initial conditions Supplementary Eq. (23) and boundary conditions Supplementary Eqs. (19)-(20). We solve this problem numerically using finite difference schemes, using standard centered finite differences for spatial derivatives (including in the boundary conditions) and the (implicit) backwards Euler method for temporal derivatives. We subdivide the spatial domain into N intervals of equal length $\Delta r = (h_l - d_l)/(2N)$ by the set of $N+1$ spatial points

$$r_i = \frac{d_l}{2} + i \times \Delta r, \qquad \text{for} \quad i = 0, 1, 2, ..., N$$

so that $r_0 = d_l/2$ and $r_N = h_l/2$. Similarly, we subdivide the temporal domain into M intervals of equal length $\Delta t = T/M$ by the set of $M+1$ temporal points

$$t_j = j \times \Delta t, \qquad \text{for} \quad j = 0, 1, 2, ..., M$$

so that $t_M = T$. We then denote the numerical approximations at selected temporal (j) and spatial (i) points as

$$C_i^j = C(t_j, r_i) \qquad B_i^j = B(t_j, r_i) \qquad I_i^j = I(t_j, r_i).$$

Discretization of the differential equations Below, we will use Δr^2 to denote $(\Delta r)^2$. Centering at $r = r_i$, we apply the finite differences to the differential equations, which gives

$$\frac{C_i^j - C_i^{j-1}}{\Delta t} = D\frac{C_{i+1}^j - 2C_i^j + C_{i-1}^j}{\Delta r^2} + D\frac{C_{i+1}^j - C_{i-1}^j}{2r_i\Delta r} - \frac{k_{\text{on}}C_r}{\varepsilon}C_i^j + k_{\text{off}}B_i^j$$

$$\frac{B_i^j - B_i^{j-1}}{\Delta t} = \frac{k_{\text{on}}C_r}{\varepsilon}C_i^j - k_{\text{off}}B_i^j - k_{\text{int}}B_i^j$$

$$\frac{I_i^j - I_i^{j-1}}{\Delta t} = k_{\text{int}}B_i^j$$

which upon rearrangement leads to

$$D\left(-\frac{\Delta t}{\Delta r^2}+\frac{\Delta t}{2r_i\Delta r}\right)C_{i-1}^j+\left(1+\frac{2\Delta tD}{\Delta r^2}+\frac{k_{\text{on}}C_r\Delta t}{\varepsilon}\right)C_i^j+D\left(-\frac{\Delta t}{\Delta r^2}-\frac{\Delta t}{2r_i\Delta r}\right)C_{i+1}^j-k_{\text{off}}\Delta tB_i^j=C_i^{j-1}$$

$$\left(1+k_{\text{off}}\Delta t+k_{\text{int}}\Delta t\right)B_i^j-\frac{k_{\text{on}}C_r\Delta t}{\varepsilon}C_i^j=B_i^{j-1}$$

$$I_i^j-k_{\text{int}}\Delta tB_i^j=I_i^{j-1}$$

From the known initial conditions ($j=0$), we can use these equations iteratively to calculate numerical solutions up to the final time $j=M$. These equations in their current form are valid for $i=1,...,N-1$. However, the equation for C centered at $d_l/2$ requires the knowledge of non-existing r_{-1} and that centered at r_N requires the knowledge of non-existing r_{N+1}. Therefore, these two equations must be modified using information coming from the boundary conditions.

Boundary conditions At the boundary points, we introduce ghost points at which the values are estimated from the discretized boundary conditions. Introducing auxiliary spatial points outside the domain, the discretized boundary condition at $r=r_N$ reads

$$\frac{C_{N+1}^j-C_{N-1}^j}{2\Delta r}=0$$

which gives

$$C_{N+1}^j=C_{N-1}^j.$$

This can be substituted back into the corresponding ($i=N$) equation for C and we get

$$-\frac{2\Delta tD}{\Delta r^2}C_{N-1}^j+\left(1+\frac{2\Delta tD}{\Delta r^2}+\frac{k_{\text{on}}C_r\Delta t}{\varepsilon}\right)C_N^j-k_{\text{off}}\Delta tB_N^j=C_N^{j-1}.$$

Similarly, the discretized boundary condition at $r=r_0$ reads

$$\frac{C_1^j-C_{-1}^j}{2\Delta r}=-\frac{P}{D}\left(C_{\text{plasma}}^j-\frac{C_0^j}{\varepsilon}\right)$$

from which we conclude

$$C_{-1}^j=C_1^j+\frac{2\Delta rP}{D}\left(C_{\text{plasma}}^j-\frac{C_0^j}{\varepsilon}\right).$$

Substituting this back into the appropriate ($i=0$) equation for C

$$D\left(-\frac{\Delta t}{\Delta r^2}+\frac{\Delta t}{2r_0\Delta r}\right)C_{-1}^j+\left(1+\frac{2\Delta tD}{\Delta r^2}+\frac{k_{\text{on}}C_r\Delta t}{\varepsilon}\right)C_0^j+D\left(-\frac{\Delta t}{\Delta r^2}-\frac{\Delta t}{2r_0\Delta r}\right)C_1^j-k_{\text{off}}\Delta tB_0^j=C_0^{j-1}$$

we get

$$\left(1+\frac{2\Delta tD}{\Delta r^2}+\frac{k_{\text{on}}C_r\Delta t}{\varepsilon}+\frac{2\Delta tP}{\Delta r\varepsilon}\left(1-\frac{\Delta r}{2r_0}\right)\right)C_0^j-\frac{2\Delta tD}{\Delta r^2}C_1^j-k_{\text{off}}\Delta tB_0^j=C_0^{j-1}+\frac{2\Delta tP}{\Delta r}\left(1-\frac{\Delta r}{2r_0}\right)C_{\text{plasma}}^j$$

Formulating the linear system $Ax=b$ Thus, for every known solution (C,B,I) at time $j-1$, we find the solution at time j by solving a linear system of algebraic equations of the form

$$\boldsymbol{A}\boldsymbol{x}=\boldsymbol{b},$$

where we wish to find a vector

$$\boldsymbol{x}=\left(C_0^j,C_1^j,...,C_N^j,B_0^j,B_1^j,...,B_N^j,I_0^j,I_1^j,...,I_N^j\right)$$

given a vector

$$\boldsymbol{b} = \left(C_0^{j-1} + \frac{2\Delta t P}{\Delta r}\left(1 - \frac{\Delta r}{2r_0}\right) C^j_{\text{plasma}}, C_1^{j-1}, C_2^{j-1} ..., C_N^{j-1}, B_0^{j-1}, B_1^{j-1}, ..., B_N^{j-1}, I_0^{j-1}, I_1^{j-1}, ..., I_N^{j-1} \right)$$

and $\boldsymbol{A}$ is a block matrix with 3×3 blocks of equal size, $(N+1) \times (N+1)$ (size $3(N+1) \times 3(N+1)$ in total), which reads

$$\boldsymbol{A} = \begin{pmatrix} \boldsymbol{A_{11}} & \boldsymbol{A_{12}} & \boldsymbol{A_{13}} \\ \boldsymbol{A_{21}} & \boldsymbol{A_{22}} & \boldsymbol{A_{23}} \\ \boldsymbol{A_{31}} & \boldsymbol{A_{32}} & \boldsymbol{A_{33}} \end{pmatrix}. \tag{26}$$

Detailing the blocks, we have

$$\boldsymbol{A_{13}} = \boldsymbol{A_{23}} = \boldsymbol{A_{31}} = \boldsymbol{0} \text{ (zero matrix)},$$

$$\boldsymbol{A_{33}} = \boldsymbol{I} \text{ (identity matrix)},$$

$$\boldsymbol{A_{12}} = -k_{\text{off}} \Delta t \boldsymbol{I},$$

$$\boldsymbol{A_{21}} = -\frac{k_{\text{on}} C_r \Delta t}{\varepsilon} \boldsymbol{I},$$

$$\boldsymbol{A_{22}} = \left(1 + \left(k_{\text{off}} + k_{\text{int}}\right) \Delta t\right) \boldsymbol{I},$$

$$\boldsymbol{A_{32}} = -k_{\text{int}} \Delta t \boldsymbol{I}$$

and

$$\boldsymbol{A_{11}} = \begin{pmatrix} a & b & 0 & \cdots & \cdots & 0 \\ c_1 & d & e_1 & \ddots & & \vdots \\ 0 & c_2 & d & e_2 & \ddots & \vdots \\ \vdots & \ddots & \ddots & \ddots & \ddots & 0 \\ \vdots & & \ddots & c_{N-1} & d & e_{N-1} \\ 0 & \cdots & \cdots & 0 & b & d \end{pmatrix}$$

where

$$a = 1 + \Delta t \left\{ \frac{k_{\text{on}} C_r}{\varepsilon} + \frac{2}{\Delta r^2}\left[D + \frac{P \Delta r}{\varepsilon}\left(1 - \frac{\Delta r}{2r_0}\right)\right]\right\}$$

$$b = -\frac{2\Delta t D}{\Delta r^2}$$

$$c_i = \frac{\Delta t D}{\Delta r^2}\left[-1 + \frac{\Delta r}{2r_i}\right]$$

$$d = 1 + \Delta t \left[\frac{2D}{\Delta r^2} + \frac{k_{\text{on}} C_r}{\varepsilon}\right]$$

$$e_i = -\frac{\Delta t D}{\Delta r^2}\left[1 + \frac{\Delta r}{2r_i}\right]$$

for $i = 1, 2, ..., N-1$. In MATLAB, we then solve $\boldsymbol{Ax} = \boldsymbol{b}$ for unknown vector $\boldsymbol{x}$ using "x=linsolve(A,b)".

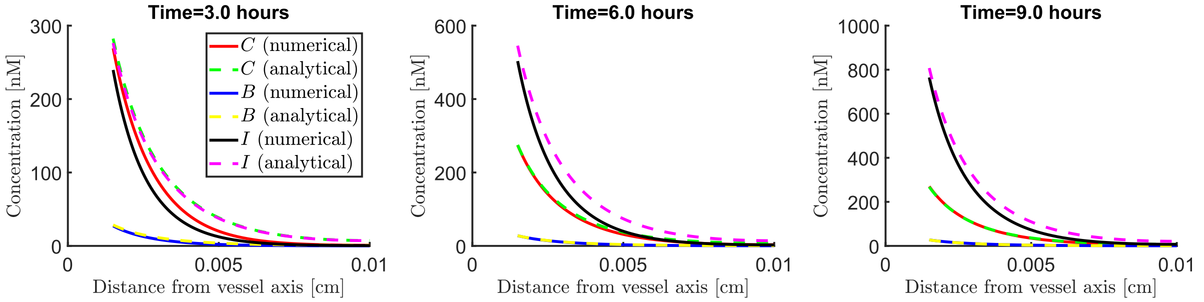

Fig C. Comparison of solution profiles as functions of the radial coordinate r at early times $t = 2$, 4 and 6 hours after the bolus injection.

Validity of the new approximation

Finally, we need to check whether the analytical solution Supplementary Eq. (25) provides a good approximation to the numerical solution of the full problem described in the previous section on the therapeutically relevant timescales (days to weeks). Using default parameter values from Table A and representative values of inter-vessel distance $h_l = 200\mu$m and vessel diameter $d_l = 30\mu$m, we find that early (3 hours) after the bolus injection, the reduced-order model gives a poor approximation to the full numerics (see Fig C). However, after $6 - 9$ hours, the reduced-order model already provides a very good approximation for free and bound drug concentrations. Due to the initial transient behaviour, which our reduced-order model does not capture, the prediction for the internalized drug I contains significant errors hours after the bolus injection. However, these differences become negligible as early as 1 day after the bolus injection (see Fig D). Moreover, the reduced-order model performs remarkably well across the parameter space spanned by the inter-vessel distance h_l and the vessel diameter d_l. As our analysis in the main body of this manuscript predicted, the largest errors occur for very large values of the vessel diameter and very small values of inter-vessel distance; however, 1 day after bolus injection, even these differences become small.

Estimation errors accumulated over the initial transient period become negligible especially over the timescale of weeks, as evidenced in Fig E (using representative model parameters). Note that as the plasma concentration decreases exponentially to 0, the amount of internalized drug increases at a declining rate. In summary, these figures confirm that the reduced-order model provides a useful approximation to the full-model behaviour on the timescales of interest (days to weeks).

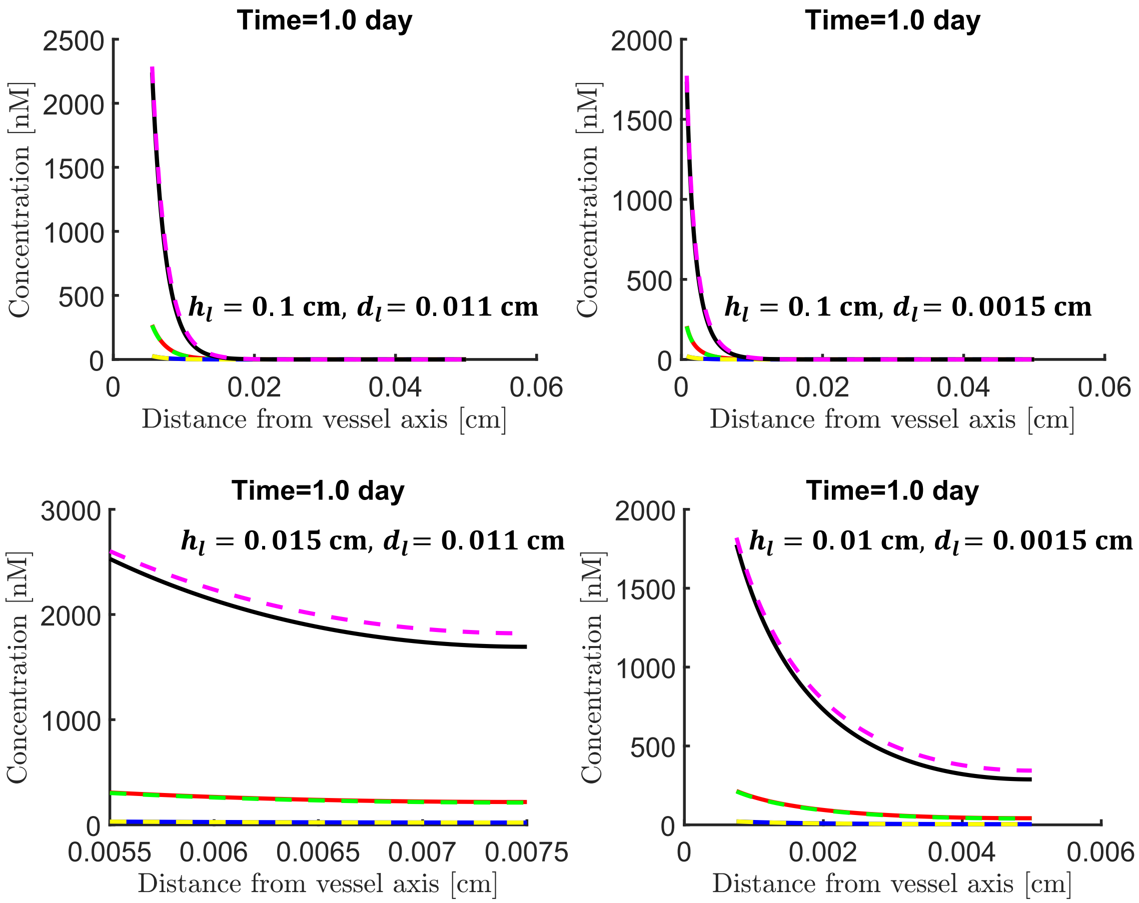

Fig D. Comparison of solution profiles as functions of the radial coordinate r, 1 day after the bolus injection for different combinations of inter-vessel distance h_l and vessel diameter d_l. The same legend as in Fig C applies.

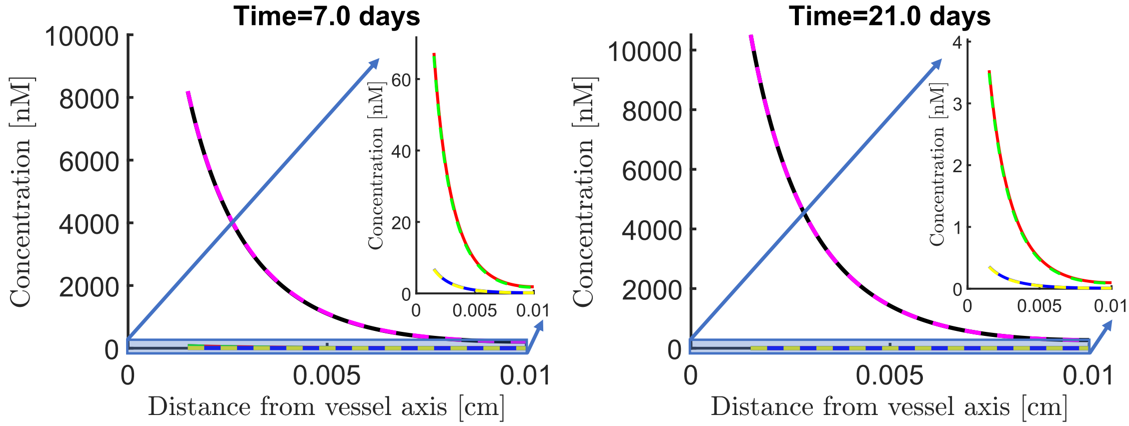

Fig E. Comparison of solution profiles as functions of the radial coordinate r at times $t = 7$ and 21 days after the bolus injection. Insets on the right of each panel document the agreement for free drug concentration C and the bound drug B. The same legend as in Fig C applies.

Efficacious T-DM1 at IC50 of 6.8 nM

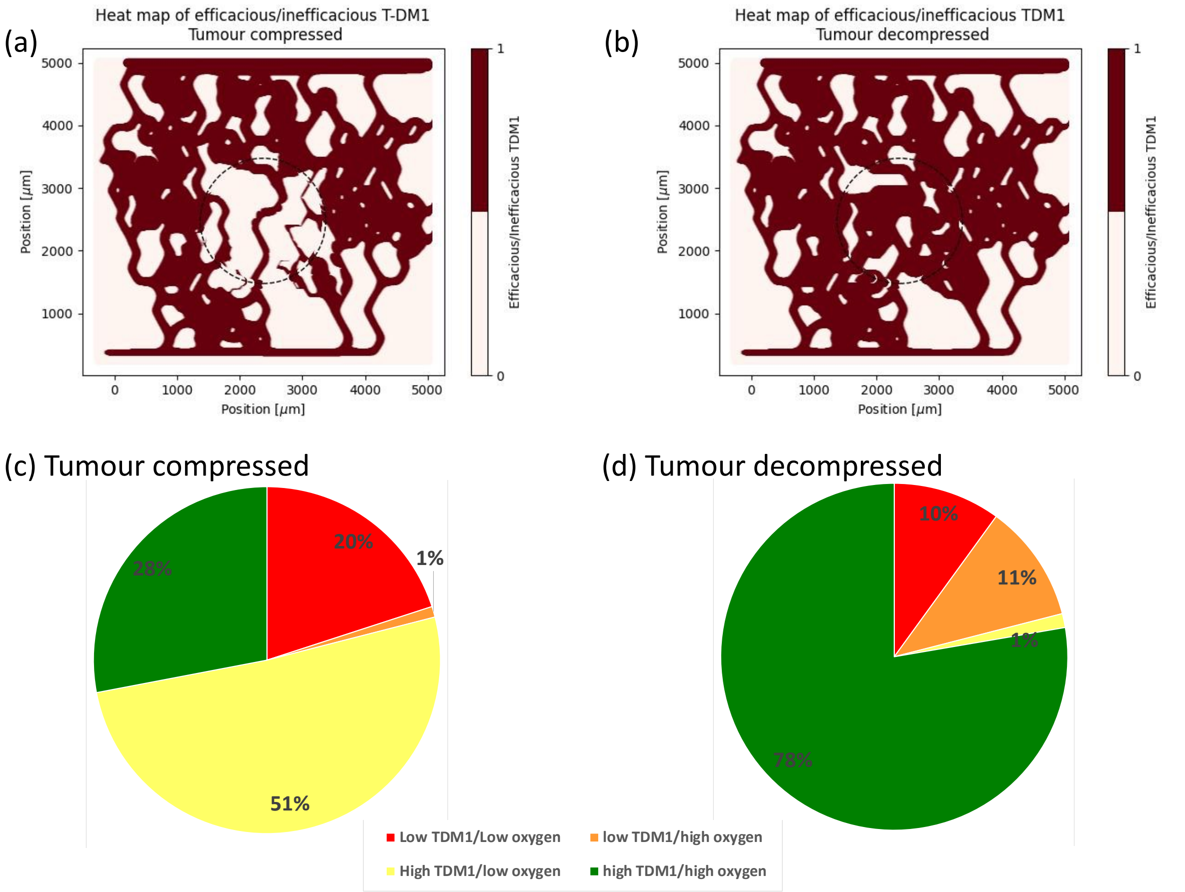

Fig F. (a) Shows efficacious T-DM1 (oxygen above 8mmHg and T-DM1 above 6.8 nM, both required for internalisation and killing cells, respectively) in tumour compressed simulation. (b) Shows efficacious T-DM1 in tumour decompressed simulation. (c) Shows fraction of tissue in core region corresponding to sufficient/insufficient oxygen/T-DM1 in tumour compressed (c) and tumour decompressed (d) cases.

Supplementary References

1. Secomb, T. W. Blood Flow in the Microcirculation. *Annual Review of Fluid Mechanics* **49,** 443–461 (2017).
2. Enjalbert, R., Krüger, T. & Bernabeu, M. O. Effect of vessel compression on blood flow in microvascular networks and its implications for tumour tissue hypoxia. *Communications Physics* **7** (Feb. 2024).
3. Pries, A. R., Neuhaus, D. & Gaehtgens, P. Blood viscosity in tube flow: Dependence on diameter and hematocrit. *American Journal of Physiology - Heart and Circulatory Physiology* **263** (1992).
4. Pries, A. R. & Secomb, T. W. Microvascular blood viscosity in vivo and the endothelial surface layer. *American Journal of Physiology - Heart and Circulatory Physiology* **289,** 2657–2664 (2005).
5. Pries, A. R., Secomb, T. W., Gaehtgens, P. & Gross, J. F. Blood flow in microvascular networks. Experiments and simulation. *Circulation research,* 826–834 (1990).
6. Lorthois, S., Cassot, F. & Lauwers, F. Simulation study of brain blood flow regulation by intra-cortical arterioles in an anatomically accurate large human vascular network: Part I: Methodology and baseline flow. *NeuroImage* **54,** 1031–1042 (Jan. 2011).
7. Fredrich, T., Welter, M. & Rieger, H. Tumorcode: A framework to simulate vascularized tumors. *European Physical Journal E* **41** (Apr. 2018).
8. Arvanitis, C. D., Askoxylakis, V., Guo, Y., Datta, M., Kloepper, J., Ferraro, G. B., Bernabeu, M. O., Fukumura, D., McDannold, N. & Jain, R. K. Mechanisms of enhanced drug delivery in brain metastases with focused ultrasound-induced blood–tumor barrier disruption. *Proceedings of the National Academy of Sciences* **115,** E8717–E8726 (2018).
9. Poon, K. A., Flagella, K., Beyer, J., Tibbitts, J., Kaur, S., Saad, O., Yi, J.-H., Girish, S., Dybdal, N. & Reynolds, T. Preclinical safety profile of trastuzumab emtansine (T-DM1): mechanism of action of its cytotoxic component retained with improved tolerability. *Toxicology and applied pharmacology* **273,** 298–313 (2013).
10. Bordeau, B. M., Abuqayyas, L., Nguyen, T. D., Chen, P. & Balthasar, J. P. Development and Evaluation of Competitive Inhibitors of Trastuzumab-HER2 Binding to Bypass the Binding-Site Barrier. *Frontiers in Pharmacology* **13** (Feb. 2022).
11. Girish, S., Gupta, M., Wang, B., Lu, D., Krop, I. E., Vogel, C. L., Burris, H. A., LoRusso, P. M., Yi, J. H., Saad, O., Tong, B., Chu, Y. W., Holden, S. & Joshi, A. Clinical pharmacology of trastuzumab emtansine (T-DM1): An antibody-drug conjugate in development for the treatment of HER2-positive cancer. *Cancer Chemotherapy and Pharmacology* **69,** 1229–1240 (May 2012).
12. Thurber, G. M. & Weissleder, R. A systems approach for tumor pharmacokinetics. *PLoS ONE* **6** (2011).
13. Grogan, J. A., Connor, A. J., Markelc, B., Muschel, R. J., Maini, P. K., Byrne, H. M. & Pitt-Francis, J. M. Microvessel chaste: an open library for spatial modeling of vascularized tissues. *Biophysical Journal* **112,** 1767–1772 (2017).
