# Supplementary material for: Abnormal vasculature reduces overlap between drugs and oxygen in a tumour computational model: Implications for therapeutic efficacy
Source: PLoS Comput Biol. 2025 Dec 22;21(12):e1013801. doi: 10.1371/journal.pcbi.1013801 (PMC12758810; doi:10.1371/journal.pcbi.1013801)
Supplement: Attachment — Submitted filename: respone_to_reviewers.pdf [file pcbi.1013801.s002.pdf]

Response letter to reviewer MS # PCOMPBIOL-D-25-00224
Abnormal vasculature reduces overlap between drugs and oxygen in a tumour computational model: implications for therapeutic efficacy

May 2025

We thank the reviewer for their thorough and constructive comments which have resulted in an improved manuscript. In this letter we address point-by-point the review received on our manuscript entitled "Abnormal vasculature reduces overlap between drugs and oxygen in a tumour computational model: implications for therapeutic efficacy" submitted for consideration to PLOS Computational Biology under manuscript reference number PCOMPBIOL-D-25-00224. The questions and comments of the reviewers are in red, whereas our responses are in black. Changes in the updated manuscript are in blue.

1 Reviewer 1

Reviewer 1 (Remarks to the Author):

In this manuscript, the authors investigate the effect of oxygen overlap on anticancer drugs that require sufficient oxygen to be effective, such as T-DM1, using a computational model that simulates both compressed and uncompressed tumor vessels. The overall presentation and flow of the manuscript can be improved. Below are several detailed comments and suggestions for revision.

1. The introduction should include an overview of the tumor microenvironment (TME) and various normalization strategies. This addition would help readers understand how compressed vessels in tumors reduce oxygenation and impair the efficacy of oxygen-dependent anticancer drugs such as T-DM1. Moreover, the manuscript should describe normalization strategies that decompress the tumor vasculature, thereby improving oxygenation and drug efficacy.

We thank the reviewer for the suggestion and have added a paragraph in the introduction to discuss normalisation strategies, and why they are relevant to this work (lines 33–39): "Treatment strategies reprogramming the tumour microvasculature have been developed with the aim to improve tumour oxygena-

tion and drug delivery [1]. These vascular normalisation therapies modulate tumour microvascular properties, such as vascular density or permeability, followed by changes in drug delivery and tissue oxygenation depending on dosage and timing [2]. In addition, drugs inhibiting collagen synthesis can decompress vessels, improving perfusion, oxygenation and drug delivery [3, 4]. The ability to reprogramme the microvasculature asks the question of how microvascular abnormality, and its modulation, has an effect on drug and oxygen overlap from a mechanistic point of view.".

Implications of this work with regards to normalisation were already in the discussion, which were slightly amended (lines 281–286).

2. A presentation of the most recent models that simulate tumor vasculature, along with a comparison to the current model, would clarify the need to simulate oxygen dynamics and the performance of oxygen-dependent drugs like T-DM1 simultaneously.

We thank the reviewer for the suggestion. In the methods section, we have directed the interested reader to the relevant review papers (lines 59–62), and emphasised that the chosen model reproduces the desired preperties for our study (lines 65–67).

3. The manuscript currently presents both the control (healthy) and tumor vasculatures with similar abnormal structures. In other words, the control case represents decompressed tumor vessels, while the tumor case represents compressed tumor vessels. To avoid confusion, I suggest replacing the term "control" or "healthy" with "normalized-decompressed tumor vasculature" and using this definition consistently throughout the manuscript. This scenario could be discussed within the context of TME normalization and extracellular matrix (ECM) normalization.

We thank the reviewer for the suggestion and have renamed the models "tumour model" and "tumour decompressed model". This has been implemented throughout the manuscript and the figures have been updated (these changes have not been highlighted).

4. Reordering Figures 2a and 2b so that the tumor's compressed vasculature is presented first, followed by the normalized-decompressed vasculature, would improve the manuscript's flow. Similarly, consider reordering Figures 3a and 3b.

We thank the reviewer for the suggestion, and the Figures have been reordered, and also renamed according to comment 3.

5. Adding a comparison case featuring normal (well-organized) vasculature with decompressed vessels would enhance the manuscript's results. This scenario can also be framed within the context of TME normalization, combining ECM and vascular normalization (see ref. "Combining two strategies to improve perfusion and drug delivery in solid tumors," DOI: 10.1158/0008-5472.CAN-13-1989).

We apologise for a lack of clarity in the initial manuscript. The microvascular

network was generated as a healthy network in order to study vessel compression in the absence of other vascular abnormalities (clarification in lines 65–67). We have not modelled other vascular normalisation or other abnormalities as this would lead to modelling difficulties, such as changes in permeability coefficients for T-DM1 or modelling of red blood cell flow due to those other vascular abnormalities. In the discussion, we address this limitation and propose future work combining experiments and modelling to tackle this more complex problem (lines 272–280).

6. The units for receptor concentration and drug concentration must be consistent for comparative purposes in Table S1 and Figures S2–S4. In instances where the drug-bound complex concentration approaches the receptor concentration, the kinetics described in Equation 2 may no longer be valid due to the assumption of a constant receptor concentration. The authors should discuss this issue and clarify whether the concentration of drug-bound complexes ever reaches that of the receptors. In Figure S4, only the internalized drug appears to reach that level.

The units for the receptor concentration in Supplementary Table 1 have been converted to nM for comparative purposes with the Supplementary Figures.

Equation 2 shows that the receptor concentration is only relevant for the binding/unbinding of the free drug to the cell surface, as described in lines 104–107. As the free drug concentration in the plasma (810 nM) is much lower than the receptor concentration (10^4 nM), it should not be a limiting factor for the model, as quantified by the non-dimensional number in Supplementary Equation 17.

7. In the "Processing results" subsection, the drug concentration is denoted as C_{T-DM1}, whereas it was previously defined simply as C. Please use consistent terminology for all variables and parameters in both the manuscript and supplementary materials. Additionally, please explain why the overlap index is calculated based on the free drug rather than the bound or internalized drug. Based on the findings reported in (https://doi.org/10.1158/1541-7786.MCR-19-0856), hypoxia affects drug internalization. Thus, it might be appropriate to calculate the overlap index using the bound drug along with oxygen levels. The authors should provide calculations for both free and bound drug and include one set in the main manuscript and the other in the supplementary material.

We apologise for the inconsistency in notation, and have uniformised it throughout the manuscript and supplementary material.

The cited work (https://doi.org/10.1158/1541-7786.MCR-19-0856) shows two key findings: that the IC_{50} of free T-DM1 is increased under hypoxic conditions, and that the mechanism for the decreased efficacy of T-DM1 under hypoxic conditions is due to a change in the drug internalisation. We choose the free drug concentration as our key metric of comparison to evaluate the efficacy of the drug, as the change in IC_{50} is shown for the free drug and not the internalised concentration. This has been clarified in the text (lines 134-135). In addition, while the paper shows that it is the internalisation process that is changed,

it does not quantify the temporal dynamics of the change in internalisation, which would be a requirement for the reaction kinetics in the reaction-diffusion equation we solve.

8. For the statement, “The distribution of oxygen concentrations in the tumour model and control model are plotted in Figure 2d, showing a lower mean and wider distribution in the tumour model compared to the control model (difference is statistically significant),” the authors need to specify the statistical test used to compare the distributions and their means, as well as the significance level. They should also indicate whether these findings apply to both the core and the periphery of the tumor. In my opinion, the distributions in the periphery appear similar aside from a scaling factor; please review Figure 2d and revise accordingly. More detailed information is also needed for the statement regarding the overlap index, “While the overlap index falls from a mean of 0.18 ± 0.21 in the control case to 0.09 ± 0.12 in the tumour model, the difference is statistically significant.”

The statistical test used and the threshold for significance are detailed in the methods section (lines 144–147).

We apologise for the lack of clarity with regards to the oxygen concentration described in this section. An amendment to the text has been made to clarify that the difference is in the core of the network (lines 170 and 172–173). The periphery is described in the following paragraph in the manuscript. The periphery is indeed similar in mean, which is why the effect size is reported, to emphasise that the difference achieves statistical significance due to the large number of points sampled, but it is modest in magnitude. Finally, the violin plot in Figure 2 did show a scaling difference, which has now been amended in the Figure.

9. The model reduction for T-DM1 transport, along with Figures S2–S4, are not mentioned in the results section. This omission should be addressed.

We apologise for the ommission, and have referenced the figures in the methods (line 126) and added a mention of the model reduction in the main text (line line 190).

10. The manuscript should clearly state where all quantified values are derived (e.g., the entire computational domain, the periphery, or the core). For instance, “As the tumour compressed vessels introduce haematocrit heterogeneity in the microvascular networks, the tumour tissue oxygenation was found to be more heterogeneous than the control, with a hypoxic fraction increasing from 11.5% to 71.6%. We then showed that due to the higher fraction of hypoxia in the tumour tissue model, there is a lower fraction of efficacious T-DM1 (28% compared to 82% in a control model) because internalization is impeded in hypoxic conditions,” it is not clear where these measurements were taken.

We apologise for the lack of clarity. The processing results section of the manuscript has been updated with an additional paragraph to detail what the periphery and the core refer to with regards to the tissue domain to clarify

this point (lines 140–143): "The drug and oxygen concentrations are measured in two separate sections of the tissue being modelled. Firstly, the core of the tissue, which is defined as being within 1000 μm of the centre of the domain, which is the same region as the one where the blood vessels are compressed. The second, the periphery region, refers to the tissue in the remaining section of the computational domain.".

11. The authors should include a discussion of the limitations of the model and the underlying assumptions.

A paragraph has been added to the discussion section addressing the limitations and assumptions of the model (lines 242–252): "In this work we simplify the model for the T-DM1 drug transport to a quasi-steady-state model, where we assume that the timescale of the decay of the T-DM1 bolus injection is much slower than the reaction-diffusion of the drug in the tissue. This allows us to find a quasi-steady-state solution for the drug in the tissue for any fixed concentration in the blood stream, as if it were not decaying with time. We demonstrate that the quasi-steady-state model shows a good match with the numerical solution of the full model, provided that the quasi-steady-state model is not used for the early hours of the bolus injection. From 1 day onwards, the simplified model shows an excellent match with the full model, see Supplementary Figures S1–S4. In addition, the simplified model does not work well for extreme combinations of large vessels and small distances between vessels, which are not present in our network. This assumption is only valid for the T-DM1 drug that we model, and would have to be verified for other drugs that have different transport timescales [5].".

12. Although the authors have included a code availability statement, a data availability statement is missing. In accordance with the journal guidelines, all data necessary to replicate the study's findings should be made publicly available.

The code availability statement has been extended to also be a data availability statement. The results for this work have been uploaded to Edinburgh DataShare (we are awaiting for the link to the data) and can be freely downloaded.

13. Minor Correction: Replace the highlighted text "In addition, tumour 20 microvascular networks can have avascular areas that lead to hypoxic regions due to the limited diffusion 21 distance of oxygen" with "In addition, tumour microenvironment 20 can have avascular areas that lead to hypoxic regions due to the limited diffusion 21 distance of oxygen."

We thank the reviewer for having spotted the mistake, the lines have been corrected (lines 30–31).

References

1. Jain, R. K. Normalizing tumour vasculature with anti-angiogenic therapy: A new paradigm for combination therapy. *Nature Medecine* **7** (2001).
2. Stylianopoulos, T., Munn, L. L. & Jain, R. K. Reengineering the Physical Microenvironment of Tumors to Improve Drug Delivery and Efficacy: From Mathematical Modeling to Bench to Bedside. *Trends in Cancer* **4,** 292–319 (2018).
3. Chauhan, V. P., Martin, J. D., Liu, H., Lacorre, D. A., Jain, S. R., Kozin, S. V., Stylianopoulos, T., Mousa, A. S., Han, X., Adstamongkonkul, P., Popović, Z., Huang, P., Bawendi, M. G., Boucher, Y. & Jain, R. K. Angiotensin inhibition enhances drug delivery and potentiates chemotherapy by decompressing tumour blood vessels. *Nature Communications* **4** (2013).
4. Martin, J. D., Lanning, R. M., Chauhan, V. P., Martin, M. R., Mousa, A. S., Kamoun, W. S., Han, H. S., Lee, H., Stylianopoulos, T., Bawendi, M. G., Duda, D. G., Brown, E. B., Padera, T. P., Fukumura, D. & Jain, R. K. Multiphoton Phosphorescence Quenching Microscopy Reveals Kinetics of Tumor Oxygenation during Antiangiogenesis and Angiotensin Signaling Inhibition. *Clinical Cancer Research* **28,** 3076–3090 (July 2022).
5. Thurber, G. M. & Weissleder, R. A systems approach for tumor pharmacokinetics. *PLoS ONE* **6** (2011).
