# Supplementary material for: Abnormal vasculature reduces overlap between drugs and oxygen in a tumour computational model: Implications for therapeutic efficacy
Source: PLoS Comput Biol. 2025 Dec 22;21(12):e1013801. doi: 10.1371/journal.pcbi.1013801 (PMC12758810; doi:10.1371/journal.pcbi.1013801)
Supplement: Attachment — Submitted filename: response_letter.pdf [file pcbi.1013801.s003.pdf]

Response letter to reviewer MS # PCOMPBIOL-D-25-00224R1 Abnormal vasculature reduces overlap between drugs and oxygen in a tumour computational model: implications for therapeutic efficacy

October 2025

We thank the reviewers for their thorough and constructive comments which have resulted in an improved manuscript. In this letter we address point-by-point the review received on our manuscript entitled "Abnormal vasculature reduces overlap between drugs and oxygen in a tumour computational model: implications for therapeutic efficacy" submitted for consideration to PLOS Computational Biology under manuscript reference number PCOMPBIOL-D-25-00224R1. The questions and comments of the reviewers are in red, whereas our responses are in black. Changes in the updated manuscript from the first review are in blue, while changes from this review are in purple.

1 Reviewer 2

1. The authors have done a good job of addressing most of the comments from the original review(s) with the exception of some nomenclature issues. The nomenclature using 'tumour' and 'tumour compressed' is confusing. Aren't all the results for a tumour. Please use the more accepted nomenclature suggested by the reviewer. Or perhaps call them 'core uncompressed' and 'core compressed'. At places in the manuscript it seems like 'tumour' refers to only the core, other times to the entire domain.

We thank the reviewer for highlighting narrative inconsistencies in the manuscript. We have updated the nomenclature to 'core uncompressed' and 'core compressed', and have made changes throughout the manuscript (not highlighted in the marked manuscript) to reflect that change and make the distinction between the core and periphery clearer.

2. The large avascular spaces in the vasculature that we see in the 'periphery' would only be expected in the core of a tumor where stresses due to cell

proliferation have already compressed some vessels. So what difference does compression make? While Tumorcode might apply Murray's law, tumor vasculature does not satisfy Murray's law. This approximation holds applies only to healthy tissues.

We agree with the reviewer that tumor vasculature does not satisfy Murray's law. However, as highlighted in lines 65–67, we generate a healthy vascular network with Tumorcode and introduce vascular compression ourselves, allowing us to study the effect of vessel compression independently of other tumour abnormalities. Vascular compression has the effect of changing the distribution of red blood cells in the network, as described in lines 78–80. Furthermore, we hypothesise that other abnormalities in the tumour microvasculature, including deviation from Murray's law which we've now added to the discussion, would contribute and exacerbate the findings in our work, and would deserve more attention, lines 278–281 which have been updated.

3. Figure 2 and the associated text do not make sense. Is Figure 2 mislabeled? Panels b and d show that the decompressed tumor has more hypoxia in the periphery than in the core. This is to be expected given that the avascular spaces in the periphery are much larger than in the core. However, panel c shows a much larger hypoxic fraction in the core than in the periphery.

We thank the reviewer for spotting a mistake in Figure 2, and for their observations on the network characteristics, which does require additional details. We updated Figure 2 and corrected the mistake. For the network, the hypoxia in the periphery area is the result of boundary effects and some avascular areas (due to the network being generated in two-dimensions and of pruning dead-end vessels). We provide additional details of the network, lines 144–149, in the updated manuscript, and refer the reader to the supplementary material. In addition, we have restricted the periphery area to the area around the core which has the same area as the core, line 144–149 and Figure 2, which removes the boundary effect.

Interestingly, the two-dimensional network we generate reproduces a characteristic of two-dimensional networks of having a lower vascular density due to missing out on neighbouring planes [1]. This is connected to the limitations of two-dimensional networks, mentioned in question 6, and is addressed in the discussion, lines 257–260 of the updated manuscript.

4. The conclusions of the study seem obvious. Oxygen diffusion distances in most tissues are known to be about a hundred microns from the nearest well perfused vessel (as confirmed in the Figure 2) . The authors provide analysis showing that a characteristic distance for the drug diffusion, sqrt(D/keff), is about 2 mm. We should expect any tissue with avascular spaces larger than 100 microns, but less than 2 mm should fall into the category of poor overlap (low oxygen/high drug). It would be helpful to include discussion of this 'back of the envelope' analysis.

This work aims to theoretically demonstrate that haematocrit heterogeneity due to vascular compression changes tissue oxygenation, and consequently the

efficacy of oxygen-dependant drugs, which, to our knowledge, has not been done before. For T-DM1, by using the diffusion coefficient $D = 4.90 \times 10^{-2} \mu\text{m}^2\ \text{s}^{-1}$ (Supplementary Table 1) and $k_{\text{eff}} = \frac{1}{t_{\text{eff}}} = \frac{1}{.13}\text{day}^{-1} = 8.90 \times 10^{-5}\text{s}^{-1}$ (equation 5 and above in main manuscript), we obtain a characteristic diffusion length, $\sqrt{\frac{4.90 \times 10^{-2}}{8.90 \times 10^{-5}}}$ of 23.46 microns. This, in fact, corresponds to Supplementary Figure 3, plotting T-DM1 concentration against distance from vessel. While this analysis is helpful, it does not account for the vascular network, and also does not spatially inform us on where the drug is efficacious based on IC_{50} concentrations. In addition, with regards to the oxygen transport, it does not account for changes in the source term due to changes in haematocrit, which is what we are interested in to inform the overalp of the oxygen, with sufficient T-DM1.

5. Convective effects from highly permeable, tumour blood vessels creating high interstitial pressure in the tumour core may contribute to poor drug delivery. Please include discussion of this limitation.

We agree with the reviewer about the effect of these abnormalities. As our work aims to investigate vessel compression in absence of other tumour abnormalities, as mentioned in lines 65–67, we do not include them. We highlight this as a modelling choice in lines 271–272 in the updated manuscript.

6. More discussion of the limitations of using a 2-D model is warranted.

We thank the reviewer for highlighting the limitations of two-dimensional simulations as opposed to three-dimensional ones. Previous work has demonstrated, using animal model data, that two-dimensional oxygen transport simulations overestimate hypoxia as they do not capture the vesels in neighbouring planes [1]. We have added this limitation in the discussion (lines 257–260).

2 Reviewer 3

Reviewer 3 (Remarks to the Author):

It has been shown that solid stress created by tumor cells can accumulate to the extent that they compress blood and lymphatic vessels. Here, the authors combine a model of blood flow/hemorheology with diffusion models for oxygen and a drug to analyze the effects of vessel compression on drug efficacy. They conclude that vessel compression creates regions where drug penetrates, but there is insufficient oxygen for uptake and killing.

The modeling is sound, and has some innovative aspects - particularly the inclusion of blood hemodynamics and partitioning in the network. The manuscript is well written, and the conclusions warranted.

However, there are concerns about the limited novelty, given the many other models and analyses in the literature. In addition, the model is perhaps overly simplistic and the interplay between hemodynamics and vessel compression geometry were not sufficiently explored.

1. The model is solving a steady-state solution and assumes the tumor and

vasculature are not changing over time. In reality, tumor cells would be dying due to the drug and lack of oxygen, thus changing metabolism locally. In addition, new vessels can sprout into hypoxic regions relatively quickly. Furthermore, the evolution of a tumor with high solid stress in the center – such as is modeled – would result in necrosis in the central region, with little metabolism or blood flow. So the initial condition of the simulations, with viable cells throughout the domain, is not justified. A time-resolved model that considers these processes would more closely reproduce tumor dynamics and give more relevant results.

We agree with the reviewer that our model is simplified, and does not capture all of the tumor dynamics detailed in the question. However, our aim is to study the effect of vascular compression on the tranport of oxygen and the overlap of oxygen and a drug (as mentioned in lines 65–67 and 271–272). The aforementioned lines highlight that it is a modelling choice to study the effect of vessel compression independently of other vascular abnormalities

2. The main innovation is the treatment of skimming and heterogeneity of hematocrit, but these aspects are not analyzed in detail. Since plasma skimming depends on the relative vessel diameters and branch angles, a sensitivity analysis should have been done with networks with various topologies/geometries. Also, a critical assumption in the model is the effect of solid stress on vessel shape. Uniformly deforming all vessels near the center to the same elliptical shape needs further justification, since vessel compression is expected to be more heterogeneous. Again, a sensitivity analysis should be shown.

We thank the reviewer for their question. We refer the reviewer to our previous work parameterising the reduced-order model [2], which is mentioned in lines 78–80 of the manuscript. In that work, we parameterise the reduced-order model for different diameters, diameter ratios, and flowrates, to account for that heterogeneity [2]. Finally, we agree about the more heterogeneous vessel compression distribution, for simplicity we chose this aspect ratio value, a median reported in the literature [3].

3. The authors should discuss how the calculations of diffusion in 2D relate to 3D tissues, and whether this might affect the conclusions.

We thank the reviewer for highlighting the limitations of two-dimensional simulations as opposed to three-dimensional ones. Previous work has demonstrated, using animal model data, that two-dimensional oxygen transport simulations overestimate hypoxia as they do not capture the vessels in neighbouring planes [1]. We have added this limitation in the discussion (lines 257–260).

4. The assumption that the source term "strengths" are proportional to local vessel diameter needs further justification. Shouldn't this also depend on flow rates and concentrations? 5. Blood becomes more efficient at releasing oxygen when sO2 is low, but the authors apparently do not include accurate oxygen saturation dynamics. This is expected to have important effects on the predicted distributions.

We have combined questions 5 and 6 as they both pertain to the same sub-

ject, and agree with the reviewer that we have used a simplified model, and address this in lines 91–92 of the updated manuscript. In effect, previous work has shown that not accounting for flowrates or saturation dynamics can still show good agreement with experimental data [1]. In addition, previous modelling has shown that haematocrit is more important than flowrate to calculate tissue oxygenation [4]. In light of those reports, we have used a simplified model which captures, to a leading order, tissue oxygenation.

6. The comments about vascular normalization need more clarification. Normalization depends largely on changes in vascular permeability, which changes transvascular plasma transport and affects interstitial flows (see, e.g. https://doi.org/10.1371/journal.pcbi.1011131). High permeability and plasma extravasation also affects hematocrit and hemodynamics. These mechanisms were not reproduced in the presented model.

We agree with the reviewer on vascular normalisation. However, we would like to distinguish vascular normalisation from stress alleviation, as described in other works [5]. While we appreciate that both are relevant (as reflected from lines 33–39 in our introduction), this work aims to study solely vessel compression, and we isolate it from other abnormalities to have a better mechanistic understanding of the effect of vessel compression, as highlighted in lines 65–67 of the updated manuscript. We have also made changes in the discussion to emphasise that we solely study vascular compression independently of other abnormalities, lines 271–272 of the updated manuscript.

References

1. Grimes, D. R., Kannan, P., Markelc, B., Bates, R., Muschel, R. J. & Partridge, M. Estimating oxygen distribution from vasculature in three-dimensional tumor tissue. *Journal of The Royal Society Interface* **13** (2016).
2. Enjalbert, R., Krüger, T. & Bernabeu, M. O. Effect of vessel compression on blood flow in microvascular networks and its implications for tumour tissue hypoxia. *Communications Physics* **7** (Feb. 2024).
3. Fang, L., He, Y., Tong, Y., Hu, L., Xin, W., Liu, Y., Zhong, L., Zhang, Y. & Huang, P. Flattened microvessel independently predicts poor prognosis of patients with non-small cell lung cancer. *Oncotarget* **8,** 30092–30099 (2017).
4. Lücker, A., Secomb, T. W., Weber, B. & Jenny, P. The relative influence of hematocrit and red blood cell velocity on oxygen transport from capillaries to tissue. *Microcirculation* **24,** 1–12 (2017).
5. Stylianopoulos, T. & Jain, R. K. Combining two strategies to improve perfusion and drug delivery in solid tumors. *Proceedings of the National Academy of Sciences* **110,** 18632–18637 (2013).
